# Tau pathology-dependent remodelling of cerebral arteries precedes Alzheimer’s disease-related microvascular cerebral amyloid angiopathy

**DOI:** 10.1007/s00401-016-1560-2

**Published:** 2016-03-17

**Authors:** Mario Merlini, Debora Wanner, Roger M. Nitsch

**Affiliations:** Institute for Regenerative Medicine - IREM, University of Zurich, Schlieren Campus, Wagistrasse 12, 8952 Schlieren, Switzerland; Faculty of Science, University of Zurich, Winterthurerstrasse 190, 8057 Zurich, Switzerland; Center for Molecular Cardiology - Vascular Aging and Stroke, University of Zurich, Schlieren Campus, Wagistrasse 12, 8952 Schlieren, Switzerland

**Keywords:** Hyperphosphorylated tau, Collagen, Internal elastic lamina, Neutrophil elastase, Vascular smooth muscle, Cerebrovascular pathology

## Abstract

Alzheimer’s disease (AD) is characterised by pathologic cerebrovascular remodelling. Whether this occurs already before disease onset, as may be indicated by early Braak tau-related cerebral hypoperfusion and blood–brain barrier (BBB) impairment found in previous studies, remains unknown. Therefore, we systematically quantified Braak tau stage- and cerebral amyloid angiopathy (CAA)-dependent alterations in the alpha-smooth muscle actin (α-SMA), collagen, and elastin content of leptomeningeal arterioles, small arteries, and medium-sized arteries surrounding the gyrus frontalis medialis (GFM) and hippocampus (HIPP), including the sulci, of 17 clinically and pathologically diagnosed AD subjects (Braak stage IV–VI) and 28 non-demented control subjects (Braak stage I–IV). GFM and HIPP paraffin sections were stained for general collagen and elastin with the Verhoeff–van Gieson stain; α-SMA and CAA/amyloid β (Aβ) were detected using immunohistochemistry. Significant arterial elastin degradation was observed from Braak stage III onward and correlated with Braak tau pathology (*ρ* = 0.909, 95 % CI 0.370 to 0.990, *p* < 0.05). This was accompanied by an increase in neutrophil elastase expression by α-SMA-positive cells in the vessel wall. Small and medium-sized arteries exhibited significant CAA-independent α-SMA loss starting between Braak stage I and II–III, along with accumulation of phosphorylated paired helical filament (PHF) tau in the perivascular space of intraparenchymal vessels. α-SMA remained at the decreased level throughout the later Braak stages. In contrast, arterioles exhibited significant α-SMA loss only at Braak stage V and VI/in AD subjects, which was CAA-dependent/correlated with CAA burden (*ρ* = −0.422, 95 % CI −0.557 to −0.265, *p* < 0.0001). Collagen content was only significantly changed in small arteries. Our data indicate that vessel wall remodelling of leptomeningeal arteries is an early-onset, Braak tau pathology-dependent process unrelated to CAA and AD, which potentially may contribute to downstream CAA-dependent microvascular pathology in AD.

**Electronic supplementary material:**

The online version of this article (doi:10.1007/s00401-016-1560-2) contains supplementary material, which is available to authorized users.

## Introduction

Pathologic cerebrovascular alterations are prominent in Alzheimer’s disease (AD) and include smooth muscle loss, vasculitis, cerebral amyloid angiopathy (CAA) with or without microhaemorrhaging, blood–brain barrier (BBB) leakage, altered vessel wall collagen content, and alterations affecting neurovascular coupling [16, 17, 25, 39, 40]. They contribute to the genesis of the neuron-hostile environment characteristic in AD. The question remains whether these vascular alterations are merely AD-related or have other, earlier origins. For example, vessel wall pathology is observed in normal ageing [25, 26] and may also play a role in primary age-related tauopathy (PART) [13, 61]. In the latter, hyperphosphorylation of tau protein causing neurofibrillary tangle (NFT) pathology occurs in the absence of amyloid β (Aβ) accumulation [13]. NFTs are also present at the earliest neurodegenerative stages that may progress to incipient AD or fully developed AD with or without concomitant Aβ pathology [8], which is interpreted by some that PART could be a mere pre-AD stage rather than a separate neurodegenerative disorder [15]. Clear is, though, that Braak tau pathology—whether as PART, related to AD, or underlying both—is accompanied by decreased cerebral blood flow [3, 9, 41]. Further, decreased cerebral blood flow [22, 32, 36, 50] along with increased vascular resistance and impaired cerebral oxygen uptake [23, 36] and overall arterial stiffness [52] is observed in subjects with mild cognitive impairment, suggesting the existence of early vessel wall remodelling because changes in the vessel wall anatomy can affect blood flow. Arterial wall remodelling is of special interest herein since an important aspect of especially large and medium-sized muscular arteries, besides functioning as a conduit, is to cushion the cardiac pulsatile blood flow waves to such extent that the pulsations experienced by the relatively fragile arteriolar and capillary wall are largely proportional to the wall’s distension capacities. This prevents microvascular wall damage [44]. Furthermore, the cerebral arterial wall is instrumental in adequate functioning of perivascular drainage, one of the main cerebral clearance routes [60, 62]. Collagen, elastin, and vascular smooth muscle form the arterial wall triad that contributes to the regulation of these blood flow dynamics, with arterial elastin playing an important role in arterial wall recoil, affecting shape during and after vascular smooth muscle-mediated vessel constriction and dilatation.

Ageing negatively affects arterial elastin, thereby altering perfusion patterns [19, 64]. To our knowledge, it is not known whether Braak tau and/or AD pathology affects elastin in cerebral arteries, similar to the finding that perivascular tau accumulation as observed in sporadic AD subjects and in a mouse model of tauopathy can instigate pathologic vessel wall changes [5, 48, 57]. Furthermore, although previous studies have shown CAA-dependent alpha-smooth muscle actin (α-SMA) and collagen loss in cerebral arterioles of AD subjects [54, 55], quantitative analyses of such vessel wall remodelling in cerebral arteries according to Braak tau stage (called “Braak stage” henceforth) in parallel to analysis of the arterioles in the same subjects have, to our knowledge, not been extensively performed. Therefore, we systematically quantified Braak stage- and CAA-dependent and -independent changes in collagen, elastin, and α-SMA and luminal diameters of the leptomeningeal arterioles, small arteries, and medium-sized arteries surrounding the gyrus frontalis medialis (GFM) and hippocampus (HIPP), including the sulci, of non-demented control (NDCTRL) and AD subjects. Concomitantly, we investigated the potential role of neutrophil elastase in affecting elastin integrity, and perivascular tau accumulation in affecting the α-SMA content of the arterial wall at the different Braak stages. The three lead questions of this study are whether (1) remodelling of the vessel wall occurs already at early Braak stages, (2) the type of remodelling is distinct between the arterioles and arteries, and (3) the type of remodelling is Braak stage and/or CAA dependent.

## Materials and methods

### Subjects and brain material

Paraffin-embedded GFM and HIPP tissue blocks of 45 subjects with Braak stage pathology ranging from score I–VI and who were clinically and pathologically diagnosed as NDCTRL or AD subjects between 1998 and 2009 were obtained from the Netherlands Brain Bank (NBB), Netherlands Institute for Neuroscience, Amsterdam. All brain material was collected from external/non-NBB-affiliated donors for or from whom the NBB had obtained a written informed consent for a brain autopsy and the use of the material and clinical information for research purposes. The authors did not participate in the clinical and neuropathological evaluations and diagnoses of the subjects. For each subject, the NBB provided the authors with information on the antemortem cognitive status [AD (*n* = 17) or NDCTRL (*n* = 28)] as diagnosed clinically according to the clinical criteria of probable AD [14, 38]—the information thereof was provided to the NBB by the respective external neurologist(s)—and post mortem on the Braak tau stage and amyloid β (Aβ) score according to [6, 7], based on Bodian, methenamine silver, and Congo red staining performed by the NBB. The neuropathological reports contained information about both the macro- and microscopic evaluation of the brain of each subject, including the absence or presence of atrophy, specific discolouration, haemorrhages, and ventricular dilatation (macroscopic evaluation); and the presence or absence of senile and/or diffuse plaques, congophilic angiopathy, and neurofibrillary tangles (microscopic evaluation). The subjects’ demographics, neuropathological diagnoses, and clinical details are summarised in Table 1.

**Table 1:** Demographics, neuropathological diagnoses, and clinical history of the subjects analysed

Braak stage	♂/♀ (*n*)	Age at death (mean ± SD, years)	ApoE^a^	Amyloid^b^	Diagnosis	History^c,d^	Cause of death^e^
I	4/6	82.1 ± 9.6	0 (4) 3/2 (2) 3/3 (4)	O (6) A (4)	NDCTRL	Angina pectoris, COPD, CVA, DM2, hypertension	Acute myocardial infarction, cachexia, dehydration, pneumonia, sepsis
II	2/4	81.4 ± 5.7	N/A (2) 3/3 (3) 4/3 (1)	A (3) B (1) C (2)	NDCTRL	Cardiac decompensation, COPD, DM2, emphysema, hypertension	Cachexia, cardiac arrest, CVA (medium-sized), dehydration
III	4/5	84.0 ± 7.5	N/A (3) 0 (2) 3/2 (1) 3/3 (2) 4/3 (1)	A (2) B (4) C (3)	NDCTRL	Angina pectoris, atrial fibrillation, cardiac decompensation, hypertension	Cardiac arrest, CVA, sudden death
IV	3/5	91.2 ± 4.4	0 (1) 3/2 (2) 3/3 (2) 4/3 (3)	C (8)	NDCTRL (3)^f^ AD (5)^f^	Cardiac decompensation, DM2, hypertension, pneumonia	Cachexia, cardiac arrest, dehydration, uncontrolled anti-coagulation therapy
V	2/3	89.0 ± 3.1	3/2 (1) 3/3 (3) 4/3 (1)	C (5)	AD	Atrial fibrillation, cardiac decompensation, hypercholesterolaemia, hypertension	Cardiac arrest, dehydration, pneumonia
VI	2/5	89.2 ± 3.4	3/3 (1) 4/3 (3) 4/4 (3)	C (7)	AD	Angina pectoris, CVA, hypertension, myocardial infarction, vascular dementia (1 subject)	Acute myocardial infarction, cachexia, dehydration, pneumonia

*AD* Alzheimer’s disease, *CERAD* Consortium to Establish a Registry for Alzheimer’s Disease, *COPD* chronic obstructive pulmonary disease, *CVA* cerebrovascular accident, *DM2* diabetes mellitus type 2, *N/A* not available, *NDCTRL* non-demented control

^a^The number in brackets represents the number of subjects with the respective ApoE isoform

^b^CERAD score. The number in brackets represents the number of subjects with the respective CERAD score

^c^Only vascular diseases and diseases affecting the vasculature are indicated

^d^The types of medication used were similar in all Braak stage groups—except for the use of antipsychotics in the Braak stage V and VI group—and included: angiotensin-converting-enzyme (ACE) inhibitors, loop diuretics, L-type Ca^2+^ channel blockers, sulfonylurea potassium channel blockers, heparin, non-steroidal anti-inflammatory drugs (NSAIDs; mainly acetaminophen, ibuprofen, and diclofenac), salicylates (mainly acetylsalicylic acid), opiates, benzodiazepines (mainly temazepam, oxazepam, and lorazepam), HMG-CoA reductase inhibitors/statins, β2-adrenergic receptor agonists, glucocorticoids, antibiotics, diarrhoea treatment (µ-opioid receptor agonists), peripheral dopamine D2/D3 receptor antagonists (domperidone), proton pump inhibitors, digoxin, nitroglycerine, racetams, typical antipsychotics (pipamperone and haloperidol)

^e^Summary of the causes of death

^f^The number in brackets represents the number of subjects with the respective diagnosis. The three NDCTRL subjects were diagnosed as NDCTRLs on the basis of their clinical cognitive status

### Histology

All reagents listed were purchased from Sigma-Aldrich (Basel, Switzerland) unless otherwise specified. The HIPP and GFM tissue blocks obtained from the NBB were cut into 5-µm-thick sections and were mounted on SuperFrost^®^ Plus microscope slides (VWR, Dietikon, Switzerland). Detailed histological staining procedures can be found in the Supplementary Materials and Methods. Briefly, following standard deparaffinisation and rehydration steps, sections for immunohistochemical staining were treated with antigen retrieval buffer followed by co-incubation with primary goat anti-α-SMA antibody (ab21027; Abcam, Cambridge, UK) and primary mouse anti-Aβ (6E10, purified; Lucerna-Chem, Luzern, Switzerland) for 1 h at room temperature (RT). Subsequently, the sections were co-incubated with donkey anti-mouse-Alexa488 and donkey anti-goat-Cy3 antibody (both from Jackson ImmunoResearch, Suffolk, UK). HIPP and GFM sections adjacent to the ones immunostained for α-SMA and Aβ were stained for collagen and elastin using the Verhoeff–van Gieson (VVG) stain. Another series of adjacent HIPP and GFM sections was stained for neutrophil elastase (primary rabbit anti-neutrophil elastase antibody, ab21595; Abcam, Cambridge, UK) using the VectaStain^®^ Elite staining kit (ReactoLab, Servion, Switzerland) in combination with the Vector^®^ SG substrate per the manufacturer’s instructions. To compare collagen stained by the Van Gieson stain with collagen stained with a collagen IV-specific antibody, a subset of sections was incubated with a mouse monoclonal antibody against human collagen IV (M 0785; DAKO, Gløstrup, Denmark) followed by staining with a donkey anti-mouse-Cy5 antibody (Jackson ImmunoResearch, Suffolk, UK). Accumulation of phosphorylated paired helical filament tau (PHF-tau) in the perivascular space of intraparenchymal vessels was detected with an antibody against phosphorylated tau (mouse anti-phospho-PHF-tau (AT8), MN1020; ThermoFisher Scientific, Reinach, Switzerland) followed by staining with a donkey anti-mouse-Cy3 antibody (Jackson ImmunoResearch, Suffolk, UK).

### Image acquisition

**Fig. 1:**
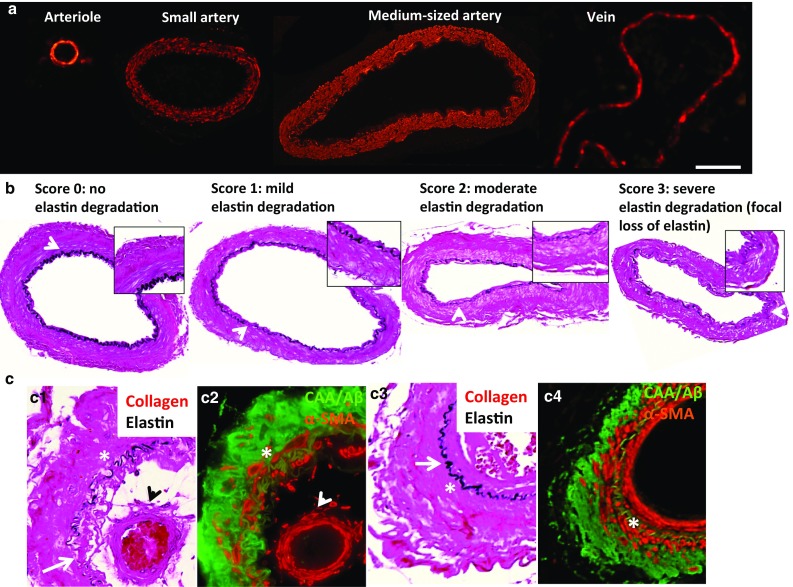
Identification of leptomeningeal arterioles and arteries and elastin degradation. Identification of the vessel types shown was based on vessel diameters as measured in alpha-smooth muscle actin (α-SMA)-stained gyrus frontalis medialis (GFM) and hippocampus (HIPP) sections (**a**). The degradation of the internal elastic lamina (**b** *arrow* indicates the internal elastic lamina/elastin) was assessed in Verhoeff–van Gieson (VVG)-stained small and medium-sized arteries according to the scoring system shown. Elastin degradation due to cerebral amyloid angiopathy (CAA) is only observed in the rare, not significant number of small and medium-sized arteries with CAA fractions >1.0 and in which CAA is present in the media [**c**, c1: VVG stain for elastin (*arrow* indicates focal loss of elastin) and collagen (*bright* *red*); *asterisk* denotes medial layer, *arrowhead* indicates double barrel formation as commonly observed for vessels with severe CAA as shown in c2 (image of adjacent section): immunohistochemical stain for amyloid β (Aβ)/CAA (*green*) and α-SMA (*red*)]. Elastin appears not to be affected by CAA in small and medium-sized arteries with adventitial CAA burden only [**c**, c3: VVG stain for elastin (*arrow* indicates preservation of elastin integrity) and collagen; *asterisk* denotes medial layer; c4 (image of adjacent section): immunohistochemical stain for Aβ/CAA and α-SMA]. *Scale bar* 100 μm

Detailed image acquisition procedures can be found in the Supplementary Materials and Methods. Microscopic images were acquired from leptomeningeal arterioles, small arteries, and medium-sized arteries surrounding the GFM and HIPP, including the sulci. Images of 10 to 15 vessels of each vessel type/brain region/subject from the VVG and immunostained HIPP and GFM sections were acquired. Differentiation between the three vessel types was made according to vessel diameter, ranging from 50 to 100 µm (arterioles), 100 to 300 µm (small arteries), and 300 to 700 µm (medium-sized arteries) (Fig. 1a). Veins and venules were not imaged and were identified by their relatively small α-SMA-to-lumen ratio. Images were acquired using the image acquisition tool of the Visiopharm software (Visiopharm, Hørsholm, Denmark). All images were acquired in a random manner blinded to subject/Braak stage.

### Image analysis: measurement of luminal diameter and Aβ, collagen, α-SMA, and neutrophil elastase vessel wall ratios

A detailed description of the analysis method can be found in the Supplementary Materials and Methods and in Supplementary Fig. 1. The green fluorescent (Aβ) and red fluorescent (α-SMA) image channels were merged in ImageJ (National Institutes of Health, Bethesda, ML, USA). Stained cells and/or cellular material, e.g. red blood cells and collagen deposits, in the lumen of both the merged fluorescent and bright field VVG- and neutrophil elastase-stained vessels were removed using ImageJ to avoid false-positive measurement hits in the subsequent quantification steps. The vessel wall fractions of Aβ (CAA), collagen, α-SMA, and neutrophil elastase and the luminal diameters were quantified using an in-house established semi-automatic quantification script written in MATLAB™ (The MathWorks, Inc., Natick, MA, USA).

### Image analysis: elastin degradation

The semi-automatic MATLAB™ quantification method mentioned above could not consistently detect local, small breakages in the arterial elastin layer (Fig. 1b, mild elastin degradation: arrowhead and inset) that were part of the elastin degradation process. Therefore, the degree of overall arterial elastin degradation was assessed in a semi-quantitative, blinded manner (see Supplementary Materials and Methods). Since no significant differences in the elastin fraction were found between CAA-affected (CAA+) and non-CAA-affected (CAA−) arteries—only in rare cases of arteries with CAA fractions >1.0 and in which CAA was present in the media (Fig. 1c: c2), which were excluded from the analysis—and between vessels in the HIPP and the GFM, the number of all small arteries and all medium-sized arteries was pooled per Braak stage.

### Statistical analyses

**Fig. 2:**
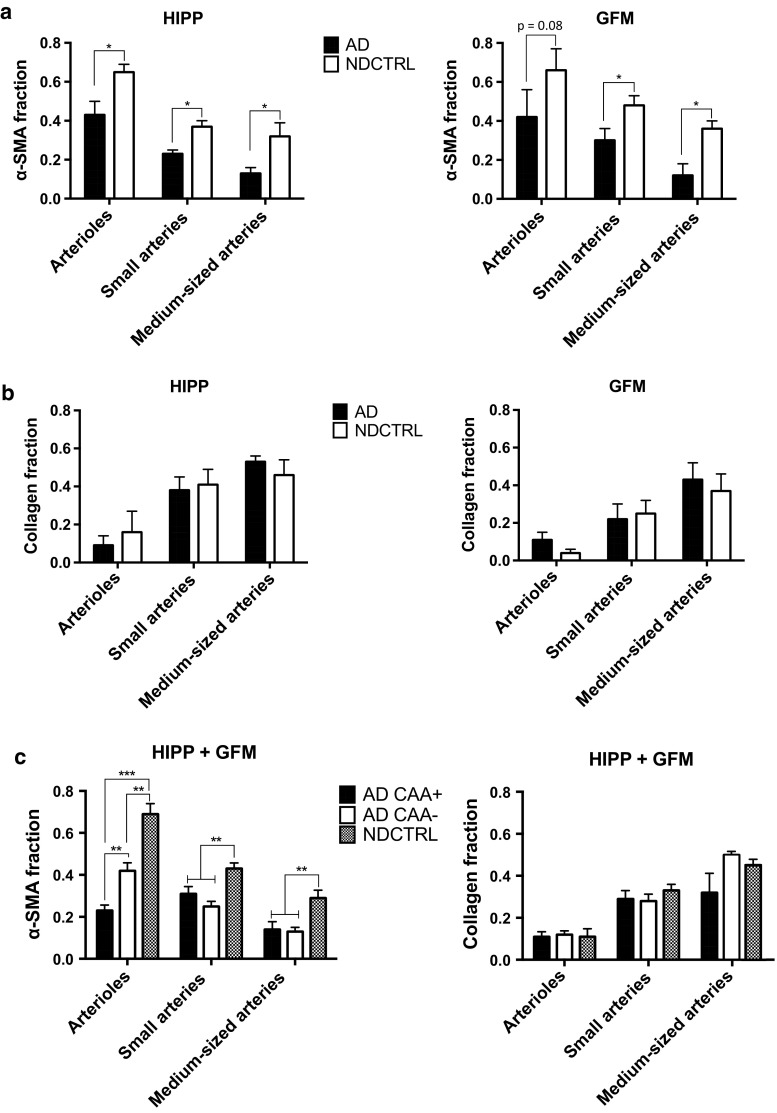
Quantification of collagen and alpha smooth muscle actin in leptomeningeal arterioles and arteries of Alzheimer’s disease and non-demented control subjects. Leptomeningeal vessels surrounding the hippocampus (HIPP) of Alzheimer’s disease (AD) subjects show a significant decrease in alpha smooth muscle actin (α-SMA) compared to vessels of non-demented control (NDCTRL) subjects (**a**). The α-SMA fraction of small and medium-sized leptomeningeal AD arteries surrounding the gyrus frontalis medials (GFM) is also significantly decreased; the α-SMA fraction of leptomeningeal AD arterioles surrounding the GFM tends to be decreased (**a**). The collagen fraction of HIPP and GFM AD vessels is not significantly different from that of their NDCTRL counterparts (**b**). Cerebral amyloid angiopathy-affected (CAA+) arterioles show exacerbation of the α-SMA loss, which is not observed for CAA+ small and medium-sized arteries (**c**). NDCTRL, AD CAA−, and AD CAA+ vessels have similar collagen fractions (**c**). The* graphs* represent the mean ± SE of 10 vessels/vessel category/subject of a total of 45 subjects; *p < 0.05, **p < 0.01, and ***p < 0.001 as determined by a two-tailed unpaired Student’s *t* test corrected for multiple comparisons (Holm–Sidak test, *α* = 0.05)

All data were expressed as the mean ± SE and were analysed using MATLAB™ (The MathWorks, Inc., Natick, MA, USA) and GraphPad Prism (GraphPad, Inc., La Jolla, CA, USA). CAA+ and CAA− blood vessels were analysed separately, except for the analyses shown in Fig. 2a, b. Blood vessels of the same type (i.e. arterioles, small arteries, or medium-sized arteries) were pooled per antemortem cognitive status of the subjects (AD or NDCTRL), CAA fraction, or Braak stage, depending on the analysis. The significance of the differences between each vessel type and the respective parameter of interest was determined using a two-tailed unpaired Student’s *t* test corrected for multiple comparisons (Holm–Sidak test, *α* = 0.05). Correlations were determined using Spearman’s correlation *ρ*_s_ (*α* = 0.05, CI 95 %, two tailed), except for the analyses of Braak pathology vs. arterial elastin degradation for which Pearson’s correlation *ρ*_p_ was used (*α* = 0.05, CI 95 %, two tailed) according to a d’Agostino–Pearson omnibus normality test.

## Results

### Alpha-smooth muscle actin is decreased in leptomeningeal arterioles and arteries of clinically and pathologically defined Alzheimer’s disease subjects

Previous studies have reported that cerebral microvessels of subjects with clinically and pathologically defined AD exhibited vascular smooth muscle loss and collagen increase [54, 55]. To confirm this finding in our study population and to understand whether leptomeningeal arterioles, small arteries, and medium-sized arteries in the same subjects might show different pathology, we analysed the α-SMA and collagen fraction in the vessel wall of pooled (i.e. leptomeningeal CAA+ and CAA−) arterioles, small arteries, and medium-sized arteries. We then grouped them according to the brain region they surrounded (HIPP or GFM) and the clinical, antemortem cognitive status of the subjects (AD or NDCTRL) (Fig. 2). All three vessel types surrounding the HIPP showed a significant decrease in the α-SMA fraction in the AD compared to NDCTRL subjects (~30–50 % decrease, *p* < 0.05) (Fig. 2a, left). The α-SMA fraction of the small and of the medium-sized arteries surrounding the GFM of AD subjects was significantly lower than that of their NDCTRL counterparts; the α-SMA fraction of arterioles surrounding the GFM showed a trend of being lower in the AD subjects than in the NDCTRL subjects (Fig. 2a, right). The collagen fractions were not significantly different between the AD and NDCTRL subjects in any of the vessel types and brain regions (Fig. 2b), although the collagen fraction of all three vessel types surrounding the GFM of both the AD and NDCTRL subjects tended to be lower than those surrounding the HIPP. Thus, overall, leptomeningeal arterioles, small arteries, and medium-sized arteries exhibited significant α-SMA loss in AD subjects whereas their vessel wall collagen content was similar between the AD and NDCTRL subjects.

The van Gieson component of the VVG stain detects collagen (bright-red stain) but lacks specificity for the different collagen types, e.g. collagen IV present in the basal lamina. Nevertheless, comparison of VVG-stained brain sections with adjacent brain sections stained with a mouse monoclonal antibody against human collagen IV showed similarity in the collagen patterns/distributions within the vessel wall between the two staining methods (Supplementary Fig. 2). The relatively high age of our study population may explain the increased collagen IV content in the basal lamina/thickening of the basal lamina compared to that in young subjects [56]. All VVG-stained vessels were carefully isolated from the acquired images and any non-vessel-related collagen was removed from the images to avoid quantification of collagen present in the leptomeninges and vessel lumen. That the van Gieson component of the VVG stain could also detect vessel wall collagen present in the medial layer can be appreciated from Supplementary Fig. 1, “Collagen fraction”.

### Analysis of CAA dependence shows CAA-dependent alpha-smooth muscle actin loss in leptomeningeal arterioles, but not in leptomeningeal arteries

To understand whether the decrease in the α-SMA fraction of the arterioles, small arteries, and medium-sized arteries in AD subjects (Fig. 2a) was related to CAA pathology and if the apparent lack of changes in the collagen fraction was due to the pooling of CAA+ and CAA− vessels (dilution effect), the three vessel types were analysed according to CAA status (CAA+ or CAA−) and antemortem cognitive status of the subjects (AD or NDCTRL) (Fig. 2c). None of the NDCTRL leptomeningeal vessels showed CAA. The α-SMA fraction of both AD CAA+ and CAA− arterioles was significantly lower than that of NDCTRL arterioles (AD CAA+ arterioles: ~70 % decrease, *p* < 0.001; AD CAA− arterioles: ~40 % decrease, *p* < 0.01) (Fig. 2c, left). Moreover, the α-SMA fraction of AD CAA+ arterioles was ~50 % lower than that of the AD CAA− arterioles, indicating that CAA exacerbated arteriolar α-SMA loss. Similar to the findings of Perry et al. [49], the α-SMA fraction of AD CAA− and CAA+ small and medium-sized arteries was significantly and equally decreased compared to that of NDCTRL small arteries and medium-sized arteries (~35–50 % decrease, *p* < 0.01; Fig. 2c, left), suggestive of a CAA-independent decrease in the arterial α-SMA fraction in the AD subjects. None of the vessels showed significant changes in their collagen fraction (Fig. 2c, right).

**Fig. 3:**
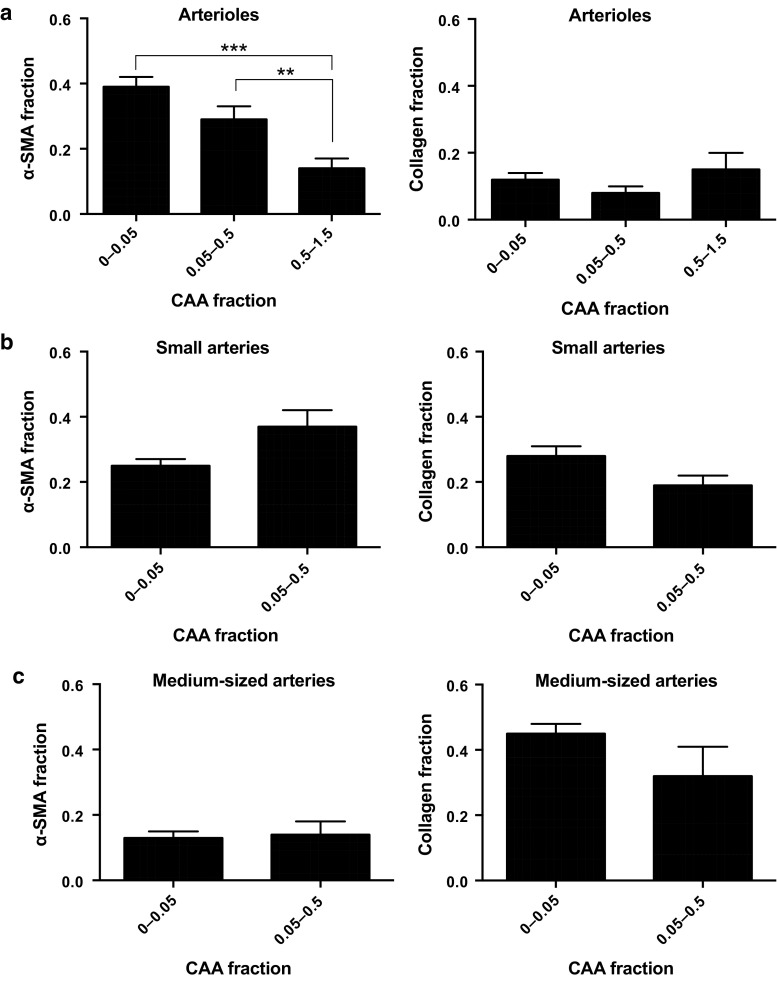
Relationship between cerebral amyloid angiopathy burden and the collagen and alpha-smooth muscle actin fraction of leptomeningeal arterioles and arteries. Leptomeningeal arterioles with the highest cerebral amyloid angiopathy (CAA) burden (“CAA fraction”) surrounding the gyrus frontalis medialis (GFM) and hippocampus (HIPP) show a significant decrease in the alpha-smooth muscle actin (α-SMA) fraction (**a**), whereas CAA burden does not affect these fractions in the leptomeningeal small arteries (**b**) and medium-sized arteries (**c**). None of the vessels show an effect of CAA burden on the collagen fraction (**a**–**c**). Mean ± SE of ~30–70 vessels/vessel category; **p < 0.01 and ***p < 0.001 as determined by a two-tailed unpaired Student’s *t* test corrected for multiple comparisons (Holm–Sidak test, *α* = 0.05)

To further analyse the CAA-independent decrease in the α-SMA fraction of the small- and medium-sized arteries in AD, and to determine whether the apparent effect of CAA on the α-SMA fraction of the arterioles in AD correlated with the degree of CAA burden, we analysed the α-SMA fraction at the different CAA fractions (Fig. 3). The vessels were categorised according to their calculated vessel CAA fraction. As shown in Fig. 3a, the α-SMA fraction was significantly decreased in arterioles with the highest CAA fraction/burden (CAA fraction 0.5–1.5 vs. 0–0.05: 63 % decreased, *p* < 0.001; CAA fraction 0.5–1.5 vs. 0.05–0.5: 51 % decreased, *p* < 0.05), and tended to be decreased in arterioles with a CAA fraction between 0.05 and 0.5 compared to those with a CAA fraction between 0 and 0.05. A Spearman’s correlation analysis revealed that arteriolar CAA burden and α-SMA loss were correlated (CAA and α-SMA: *ρ*_s_ = −0.422, 95 % CI −0.557 to −0.265, *p* < 0.0001). In line with the results found for small and medium-sized arteries of AD subjects with and without CAA (Fig. 2c, “CAA+” and “CAA−”), no significant CAA-dependent change in the α-SMA fraction was observed in either of these artery types (Fig. 3b). Further analysis revealed again that none of the vessels showed significant changes in their collagen fraction (Fig. 3, right graphs).

### Analysis of Braak stage dependence shows that leptomeningeal arteries, but not leptomeningeal arterioles, exhibit early and Braak stage-dependent alpha-smooth muscle actin loss

**Fig. 4:**
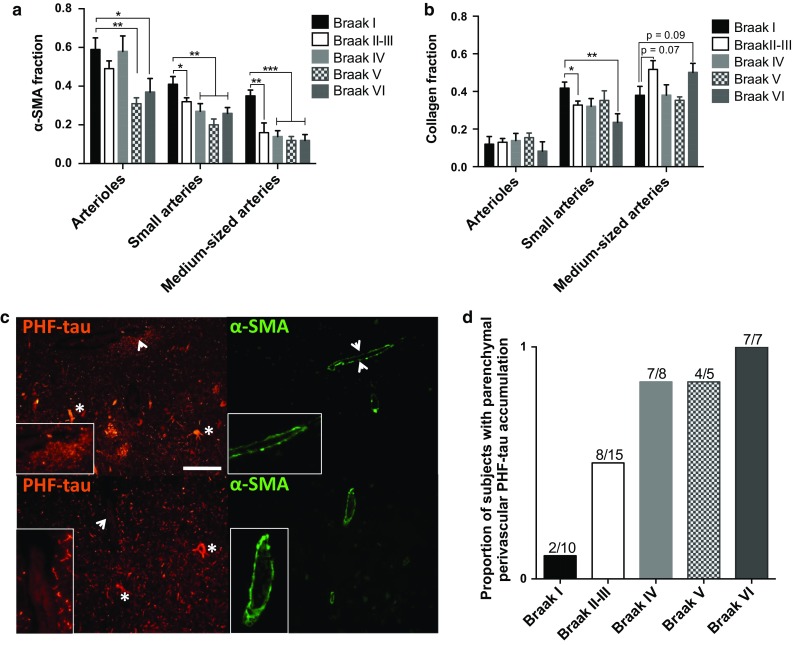
Relationship between Braak pathology, the collagen and alpha-smooth muscle actin fraction of leptomeningeal arterioles and arteries, and perivascular accumulation of phosphorylated paired helical filament tau. Small and medium-sized leptomeningeal arteries surrounding the gyrus frontalis medialis (GFM) and hippocampus (HIPP) show a significant reduction in the alpha-smooth muscle actin (α-SMA) fraction between Braak stage I and II–III, which remains reduced throughout the later Braak stages; leptomeningeal arterioles show a significant α-SMA loss only at Braak stage V and VI (**a**). The collagen fraction of only small arteries is significantly changed (i.e. reduced) with increasing Braak stage (**b**). Mean ± SE of 10 vessels/vessel category/subject of a total of 45 subjects; *p < 0.05, **p < 0.01, and ***p < 0.001 as determined by a two-tailed unpaired Student’s *t* test corrected for multiple comparisons (Holm–Sidak test, *α* = 0.05). Intraparenchymal perivascular accumulation of phosphorylated paired helical filament tau (PHF-tau) (**c** *upper panel* PHF-tau staining: area indicated by *arrowheads* is shown enlarged in *inset*; *asterisks* indicate intraneuronal PHF-tau) is accompanied by α-SMA loss (**c** *upper panel* α-SMA staining: *arrowheads* point to discontinuous α-SMA staining; enlarged in *inset*). Compare with the largely continuous, uniform α-SMA staining of a small intraparenchymal artery without perivascular PHF-tau accumulation (**c** *lower two panels*; PHF-tau staining: *arrowhead* points to absence of perivascular PHF-tau; area indicated by *arrowhead* is shown enlarged in *inset*; *asterisks* indicate intraneuronal PHF-tau). The proportion of subjects per Braak stage with parenchymal perivascular PHF-tau accumulation is increased with increasing Braak tau pathology (**d**). All images were acquired from consecutive HIPP sections of a Braak stage II subject. *Scale bar* 50 μm

The difference in CAA dependence of α-SMA loss between the arterioles and the arteries (Figs. 2, 3), and the similarities in collagen content between AD, NDCTRL, CAA-, and non-CAA-affected vessels prompted us to study whether Braak tau pathology, independent of CAA and cognitive status of the subjects, might affect the cerebrovascular α-SMA and collagen content in our study population, and whether this might also be vessel type dependent. To this end, the α-SMA and collagen fractions of arterioles, small arteries, and medium-sized arteries without CAA (“CAA−”) were categorised according to Braak stage, rather than to antemortem clinical AD or NDCTRL diagnosis (Fig. 4). A significant decrease in the α-SMA fraction was observed for small and medium-sized arteries between Braak stage I and II–III (small arteries: ~25 % decrease, *p* < 0.05; medium-sized arteries: ~50 % decrease, *p* < 0.01). Although the α-SMA fraction of both artery types was not significantly different between Braak stage II and VI (Fig. 4a), comparison of the α-SMA fraction between Braak stage I and IV, I and V, and I and VI showed a further significant decrease in the α-SMA fraction of both artery types (*p* < 0.01–0.001). Separate correlation analysis considering all Braak stages and the respective α-SMA fraction showed that these two parameters were weakly correlated in the small arteries and moderately correlated in the medium-sized arteries (*ρ*_s_ small arteries = −0.216, 95 % CI −0.317 to −0.110, *p* < 0.001; *ρ*_s_ medium-sized arteries = −0.433, 95 % CI −0.640 to −0.167, *p* < 0.001). Similar to what was found by others [54], arteriolar α-SMA loss was not Braak stage dependent, and a significant decrease in the arteriolar α-SMA fraction was observed only at Braak stage V and VI (~40–45 % decrease, *p* < 0.05–0.01) (Fig. 4a).

The collagen fraction of small arteries of Braak stage II–III and VI subjects was significantly decreased compared to that of their Braak stage I counterparts (Braak stage II–III: ~21 % decrease, *p* < 0.05; Braak stage VI: ~43 % decrease, *p* < 0.01) (Fig. 4b); Braak stage and the observed decreased collagen fraction of the small arteries were, however, only weakly correlated (*ρ*_s_ = −0.256, 95 % CI −0.390 to −0.112, *p* < 0.001). Although not significant, medium-sized arteries tended to show an increase in the collagen fraction between Braak stage I and II–III and I and VI (~25 % increase, *p* = 0.07 and *p* = 0.09) (Fig. 4b). Similar to the lack of Braak stage-dependent arteriolar α-SMA loss, the arterioles in these CAA-negative samples did not show a significant change or trend in the collagen fraction with increasing Braak stage (Fig. 4b), further confirming the Braak tau-independent origins of the microvascular pathology found in this and a previous study [54].

### Braak tau pathology does not significantly affect the wall-to-lumen ratio and the luminal diameter of leptomeningeal vessels

As described by others, CAA-affected vessels show a decrease in their wall-to-lumen ratio [29, 54]. Contrary to this, herein, we found that Braak tau pathology did not significantly alter the wall-to-lumen ratio of the arterioles, small arteries, and medium-sized arteries at all Braak stages analysed (Supplementary Fig. 3a). The average wall-to-lumen ratio at all Braak stages of the arterioles, small arteries, and medium-sized arteries was 0.37, 0.29, and 0.25, respectively, in line with the reduction in this ratio with increasing vessel size [4]. The relatively high values found for all three vessel types may be expected for vessels from aged subjects [2, 56]. The lack of a change in the arteriolar wall-to-lumen ratio with increasing Braak stage agrees with previous findings [54] and with our other results of Braak stage-independent changes in the arterioles described herein. We ascribe the unchanged wall-to-lumen ratio of the small and medium-sized arteries to the fact that we measured the overall, total thickness of the vessel wall (intima to outer layer of the adventitia) to correct for the arterial Braak stage-dependent α-SMA loss described above, which would otherwise skew these results. Thus, arterial α-SMA loss did not affect the overall, total vessel wall thickness of the arteries.

Analysis of the luminal diameter per Braak stage, however, revealed that the percentage of arterioles within the smallest luminal diameter range measured (50–75 µm) was increased at Braak stage V and VI (~20–25 % increase) although this was not significant (*p* > 0.05) (Supplementary Fig. 3b). A less pronounced shift toward decreased luminal diameters was observed for small arteries (Braak stage IV and higher) and medium-sized arteries (Braak stage II/III and higher) (Supplementary Fig. 3b). Given the relatively high α-SMA but low collagen content of specifically arterioles—i.e. low arteriolar wall stiffness—compared to arteries, arteriolar loss of α-SMA-positive cells/vascular smooth muscle cells as we found at Braak stage V and VI (Fig. 4) may explain the more profound decrease in arteriolar luminal diameter compared to that of the stiffer small and medium-sized arteries. These reductions in arteriolar luminal diameter may play a role in cerebral hypoperfusion at progressive Braak tau stages as found in previous studies [3, 9, 41]. On the other hand, the lack of a profound effect of Braak stage on luminal diameter of the leptomeningeal arteries, which are upstream of the arterioles, suggests that cerebral hypoperfusion—at least at early Braak tau stages—may be more related to altered arterial wall compliance causing alterations in perivascular drainage and blood flow regulation rather than to actual arterial luminal diameter changes.

### Accumulation of phosphorylated paired helical filament tau in the perivascular space of intraparenchymal vessels is accompanied by intraparenchymal alpha-smooth muscle actin loss and increases with Braak stage

Given that accumulation of PHF-tau has been observed in the perivascular space of both CAA-affected and -unaffected intraparenchymal vessels in subjects with sporadic AD [48, 57], we hypothesised that the CAA-independent but Braak stage-dependent loss of α-SMA in the small and medium-sized leptomeningeal arteries (Figs. 3b, c, 4a) might have been related to the perivascular drainage of toxic PHF-tau species from intraparenchymal vessels to the leptomeningeal arteries. Staining of HIPP sections of all 45 subjects for PHF-tau revealed the presence of PHF-tau in the perivascular space of intraparenchymal vessels in especially the CA1 region bordering the subiculum [33] in subjects with Braak stage II pathology and higher (Fig. 4c, Supplementary Fig. 4). The number of subjects affected by PHF-tau increased with progression of Braak tau pathology (Fig. 4d); however, the number of vessels showing this phenomenon highly varied between the subjects (on average, 5–20 vessels out of 50 vessels analysed were affected in the subjects). Perivascular PHF-tau accumulation was observed around intraparenchymal veins, arterioles, and small arteries, and was accompanied by α-SMA loss (Fig. 4c, Supplementary Fig. 4). None of the leptomeningeal vessels analysed in this study showed such perivascular PHF-tau accumulation, indicating that potential PHF-tau-related α-SMA loss in the leptomeningeal arteries might only have been caused by perivascular drainage of soluble PHF-tau species.

### Leptomeningeal arteries exhibit Braak stage-dependent elastin degradation starting at early Braak tau pathology

**Fig. 5:**
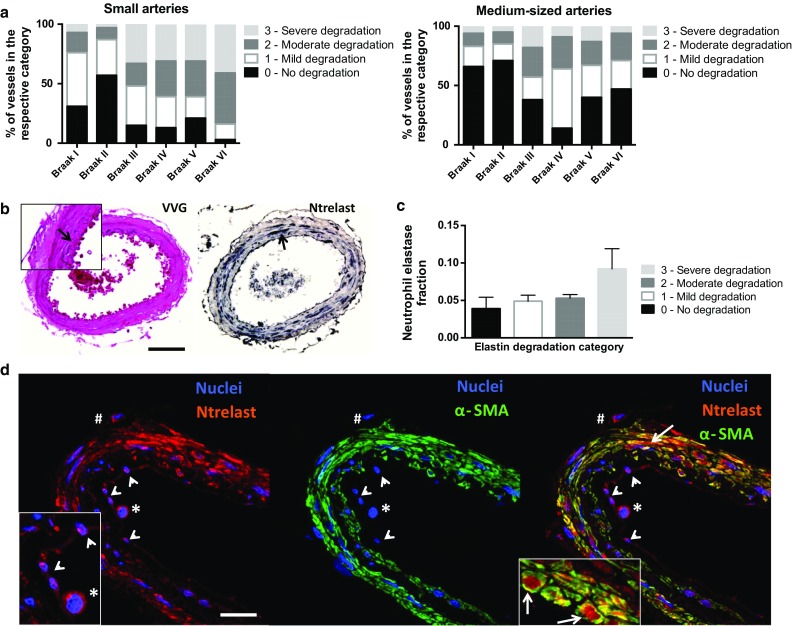
Quantification of Braak stage-dependent elastin degradation and neutrophil elastase presence in the wall of leptomeningeal arteries. Elastin in leptomeningeal arteries is degraded in a Braak stage-dependent manner, which differs between small and medium-sized leptomeningeal arteries (**a**). The percentage of small arteries with moderate (score 2) elastin degradation is increased between Braak stage II and VI; the percentage of small arteries with severe elastin degradation sharply increases between Braak stage II and III and remains at this level between Braak stage III and VI (**a** *left*). The percentage of medium-sized leptomeningeal arteries with moderate elastin degradation (score 2) is increased between Braak stage II and III and remains at this level between Braak stage III and VI (**a** *right*). The overall percentage of medium-sized arteries with severe elastin degradation is not increased between Braak stage I and VI (**a**). Severe arterial elastin degradation [**b** Verhoeff–van Gieson (VVG) stain: arrow in inset indicates focal elastin loss] tends to be accompanied by increased vessel wall neutrophil elastase fractions (**b** Ntrelast: *arrow*; **c** *bar graph*). Confocal microscopy of Ntrelast in the arterial wall reveals Ntrelast expression by α-SMA-positive cells/smooth muscle cells [**d** *right panel* co-localisation (*yellow*) and *arrows*]. Ntrelast-positive cells attached to the luminal side of the intimal layer (**d** *arrowheads* and *asterisk*) and present between the leptomeningeal layer and the adventitia (**d** *number sign*) are identified as neutrophils (Ntrelast is also a neutrophil-specific marker), and are not α-SMA-positive cells [**d** *middle panel* absence of α-SMA staining around DAPI-stained nuclei at the luminal side of the intimal layer (*arrowheads* and *asterisk*) and between the leptomeningeal layer and the adventitia (*number sign*)]. Some neutrophils have a flattened morphology (**d** *left* and *right panel*: *arrowheads*), suggestive of diapedesis. *Scale bar* 20 μm

The arterial internal elastic lamina is formed by vascular smooth muscle-derived elastin and plays an important role in vessel wall distension and recoil at normally elevated blood pressure levels [44, 58]. Elastin can be degraded enzymatically, chiefly by neutrophil- and/or vascular smooth muscle-derived neutrophil elastase, and by chronic hypertensive vessel wall stress [31, 34, 42, 43]. Importantly, vessel wall collagen governs vessel wall distension during high-pressure vessel wall stress [44, 58]. Given the significant Braak stage-dependent α-SMA loss in both the small and medium-sized arteries and collagen loss in the small arteries (Fig. 4), we hypothesised that arterial elastin might also be affected by Braak tau pathology. Indeed, quantification of arterial elastin degradation revealed a Braak stage-dependent increase in the percentage of small arteries with moderate elastin degradation (score 2, 26 % increase) between Braak stage II and VI, which was accompanied by a decrease in the percentage of small arteries without (score 0, 42 % decrease) and with mild (score 1, 32 % decrease) elastin degradation (Fig. 5a, left). However, between Braak stage I and II, the percentage of small arteries in all three elastin degradation categories was decreased, which was reflected by an increase in the percentage of vessels without elastin degradation (score 0, ~40 % increase). The percentage of small arteries with severe elastin degradation was sharply increased between Braak stage II and III (score 3, 33 % increase), and largely remained at this level at the later Braak stages. The elastin degradation in the medium-sized arteries was less severe than that observed in the small arteries, and followed a different pattern (Fig. 5a, right). The percentage of medium-sized arteries with mild and moderate elastin degradation was increased between Braak stage II and IV (score 1, 33 % increase; score 2, 16 % increase). However, the percentage of medium-sized arteries with mild elastin degradation was sharply decreased between Braak stage IV and VI (score 1, 26 % decrease), and this was accompanied by an increase in the medium-sized arteries without elastin degradation (score 0, 33 % increase) (Fig. 5a). The percentage of medium-sized arteries with moderate elastin degradation, however, remained largely stable between Braak stage III and VI (score 2, 25 and 23 %, respectively) (Fig. 5a). The percentage of medium-sized arteries with severe elastin degradation was not changed considerably between all Braak stages analysed (score 3, ~6–9 %), except at Braak stage III (score 3, 19 %) and V (score 3, 13 %) (Fig. 5a). CAA appeared to affect elastin integrity only in the rare number of small and medium-sized arteries affected heavily by CAA in which CAA was present in the media (Fig. 1c: c1 and c2), but not in the small and medium-sized arteries with adventitial CAA burden only (Fig. 1c: c3 and c4).

As shown in Table 2, correlation analysis revealed a significant positive correlation between Braak stage and moderate elastin degradation in the small arteries. However, no significant correlation between Braak stage and any of the four elastin degradation categories was observed in the medium-sized arteries. These and the above results showed that elastin degradation in small arteries progressed with Braak tau pathology, whereas elastin degradation in medium-sized arteries was less prevalent and, although it progressed between Braak stage II and III, it did not further increase in severity during the more advanced Braak stages.

**Table 2:** Correlation analysis between the number of small and medium-sized leptomeningeal arteries in the respective elastin degradation category and Braak stage

	Small arteries			Medium-sized arteries		
	*ρ*_p_^b^	CI (95 %)	*p*	*ρ*_p_^b^	CI (95 %)	*p*
Braak^a^ vs. “0”	−0.707	−0.965 to 0.246	0.116	−0.546	−0.941 to 0.476	0.262
Braak^a^ vs. “1”	−0.955	−0.995 to −0.638	0.003*	0.489	−0.535 to 0.931	0.325
Braak^a^ vs. “2”	0.909	0.370 to 0.990	0.012*	0.706	−0.247 to 0.965	0.117
Braak^a^ vs. “3”	0.792	−0.056 to 0.976	0.061	−0.041	−0.825 to 0.797	0.939

“0”, “1”, “2”, and “3” = no, mild, moderate, and severe elastin degradation, respectively

*CI* confidence interval

* Significant

^a^Braak stages included: I–VI

^b^Pearson’s correlation (*α* = 0.05, two tailed)

We postulate that the significant collagen loss with progressing Braak stage pathology specifically in the small arteries causes higher vessel wall stress and thus more pressure-induced elastin loss in these vessels than in the medium-sized arteries. This may explain the difference in elastin degradation severity between these otherwise similar artery types.

### Severe leptomeningeal arterial elastin degradation tends to be accompanied by increased leptomeningeal arterial smooth muscle neutrophil elastase expression

The enzymatic degradation of elastin referred to above is prominent in chronic obstructive pulmonary disease (COPD), where neutrophil-derived neutrophil elastase compromises the vessel wall integrity of pulmonary arteries resulting in emphysema [31]. Inhibition of neutrophil elastase alleviates elastin degradation and emphysema in COPD [1, 31], as well as vasogenic edema and neuronal loss in acute cerebral ischemia and traumatic brain injury [28, 53]. Here, severe elastin degradation in small and medium-sized arteries (Fig. 5b) tended to be accompanied by increased vessel wall neutrophil elastase fractions (Fig. 5b, c). Confocal microscopy revealed that the neutrophil elastase observed within the vessel wall was expressed by α-SMA-positive cells/smooth muscle cells (Fig. 5d, arrows in right panel) and most likely not by neutrophils (neutrophil elastase is also a specific neutrophil marker) as these were only observed attached to the luminal side of the intimal layer (Fig. 5d, asterisk and arrowheads) and between the leptomeningeal layer and the adventitia (Fig. 5d, number sign). However, extravasation of neutrophils into the vessel wall could not be ruled out, as some neutrophils attached to the luminal side of the intimal layer exhibited a flattened morphology suggestive of diapedesis (Fig. 5d, arrowheads). Thus, these results suggest a role for increased and/or aberrant neutrophil elastase expression by smooth muscle cells in severe elastin degradation in leptomeningeal arteries.

## Discussion

Herein, we found that leptomeningeal arteries show CAA-independent pathologic vessel wall remodelling that is related to early Braak tau pathology, while leptomeningeal arterioles only show significant vessel wall remodelling at late/AD Braak stages and which is CAA dependent. This suggests that early, pre-AD cerebral artery pathology unrelated to Aβ burden may contribute to the impairment of downstream arterioles in AD. Nonetheless, the following two major limitations of our study need to be considered: the relatively small population size and the exclusion of intraparenchymal vessels in the majority of our analyses. (1) Although our population size and Braak stage range were similar to those of previous studies [17, 54, 55], the majority of subjects had vascular-related morbidities, including chronic hypertension, diabetes type 2, and cardiac impairment. These factors were not biased toward a specific Braak stage (Table 1) but all of them could impact vascular health. (2) Our goal was to determine the effects of Braak tau pathology and CAA on the cerebrovasculature. Therefore, to avoid measuring these along with the (combined) effects of parenchymal Aβ plaques, neurofibrillary tangles, glial cells, and the accompanying pathologic changes in the parenchymal brain milieu on vessel wall remodelling, we had analysed only leptomeningeal vessels. Consequently, potential Braak stage- and CAA-dependent pathology of intraparenchymal vessels, except for perivascular PHF-tau accumulation, was not determined.

Our findings of Braak stage-dependent arterial elastin degradation offer insight into an understudied pathology of the cerebral arteries. Elastin loss is known to contribute to vessel tortuosity and, thereby, aberrant perfusion [34, 47]. Thus, the arterial elastin degradation we have observed may play a role in previously described impaired perivascular drainage [25–27, 59, 62] and cerebral hypoperfusion that worsens with advancing Braak stages [3, 9, 41]. Especially, reduced brain tissue oxygenation due to cerebral hypoperfusion becomes significant at Braak stage III–IV [41], similar to the moderate and severe arterial elastin degradation we found. Such arterial elastin degradation is, to our knowledge, not known to be present in relatively healthy aged individuals [19]. Given that the majority of the Braak stage IV and all of the Braak stage V and VI subjects had clinically and pathologically defined AD and that moderate and severe elastin degradation were prominent at these advanced Braak stages, elastin degradation might be an important degenerative process contributing to vessel wall pathology affecting vascular tone in AD. The trend for an increased expression of neutrophil elastase by α-SMA-positive cells in the arteries with severe elastin degradation we found points to one potential mechanism underlying the observed arterial elastin degradation, which we are currently studying in detail. Other potential mechanisms are (1) the upregulated expression and/or activity of matrix metalloproteinases (MMPs), particularly MMP-2 and MMP-9, in the vessel wall as they are known to degrade arterial elastin, are linked to mild cognitive impairment and AD, and are activated by Aβ oligomers [10, 24, 35]; and (2) altered production of vascular smooth muscle-derived arterial elastin due to the change of a contractile to a proliferative vascular smooth muscle phenotype [30, 47]. In light of the latter, elastin is known to promote the physiological contractile, non-proliferative vascular smooth muscle phenotype [30, 47]; thus, elastin loss/degradation in itself may contribute to changes in vascular smooth muscle behaviour. Important to note here is that neutrophil elastase induces both vascular smooth muscle cell apoptosis and increased vascular smooth muscle expression of pro-inflammatory chemokines causing vessel wall remodelling [12, 46]. These findings suggest that the loss of arterial α-SMA-positive cells/smooth muscle cells we describe may also be related to the cells’ increased neutrophil elastase expression.

The CAA-independent but Braak stage-dependent α-SMA loss in both artery types from Braak stage II/III onward we observed was supported by previous findings of CAA-independent vascular smooth muscle loss in the posterior cerebral artery in AD [49], and was different from the CAA dependence of the arteriolar/microvascular α-SMA loss we and others [17, 54, 55] had found. CAA causes pathologic remodelling of the vessel wall that is linked to CAA-induced hypoperfusion [11] and impaired perivascular drainage [26]. Conversely, chronic, CAA-/Aβ-independent hypoperfusion instigates microvascular CAA formation, cortical microinfarcts, and neurovascular dysfunction [16, 45], and progresses with Braak tau pathology [9, 41]. Further, while Aβ_42_-induced endothelin-1 upregulation causing vasoconstriction/hypoperfusion becomes significant only at Braak stage V–VI/in AD patients [41], as mentioned, significantly reduced cerebral tissue oxygenation can already be observed at Braak stage III–IV [41]. The finding of PHF-tau accumulation in the perivascular space of intraparenchymal vessels starting at early Braak stages we describe here and is present in AD as has been reported by others [48, 57] may suggest a tau pathology-driven mechanism underlying this hypoperfusion. Given that the intraparenchymal vessels with perivascular PHF-tau accumulation showed α-SMA loss, and that perivascular molecules have access to perivascular drainage pathways and thus leptomeningeal arteries [26, 60, 62], it is likely that toxic (oligomeric) PHF-tau species contributed to the early Braak stage arterial α-SMA loss. This notion is supported by the fact that arterial α-SMA loss, arterial elastin degradation, and intraparenchymal perivascular PHF-tau accumulation all started around Braak stage II–III, suggesting a link between the loss of vessel wall constituents important for cerebral blood flow/perivascular drainage and perivascular PHF-tau.

Yet, a potential involvement of Aβ in the early arterial wall changes observed herein cannot be ruled out. Soluble Aβ oligomeric species, for example, are already present at Braak stage I–II [37]. Even though Aβ oligomer-mediated vasoconstriction via endothelin-1 upregulation may be unlikely to occur at early, pre-AD Braak stages as mentioned above, (soluble) Aβ oligomers are cytotoxic and can be generated by vascular smooth muscle cells [20, 21]. Neuron-derived Aβ oligomers, similar to tau oligomers, are cleared along and may accumulate around the leptomeningeal arteries through the workings of the perivascular drainage system [26, 60]. Furthermore, although a clear effect of CAA on arterial elastin was not observed in this study, amyloidogenic proteins have been reported to bind and interact with elastin [18, 63] and, as mentioned, can activate MMPs, which degrade elastin.

**Fig. 6:**
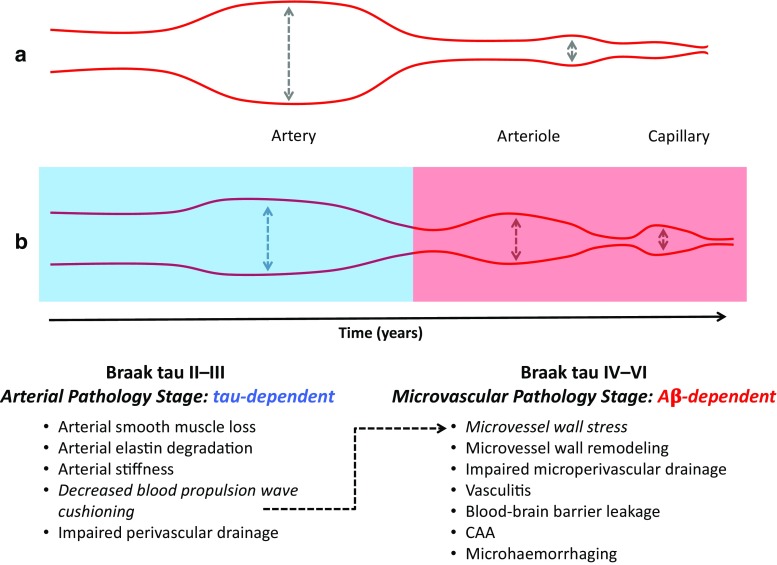
Proposed model of early Braak stage, tau pathology-dependent remodelling of cerebral arteries instigating cerebral amyloid angiopathy-related microvascular pathology. In the vascular system under healthy, physiological conditions (**a**), arteries cushion the blood propulsion wave amplitudes originating from aortic blood propulsions by distension of their vessel wall. This mechanism ensures that the blood propulsion wave amplitudes are decreased such that the ones experienced by the downstream, relatively fragile arterioles and capillaries are proportional to their small vessel wall distension capacity. Arterial elastin degradation and vascular smooth muscle loss start at early Braak tau stages (**b**), coincide with increasing (perivascular) tau pathology, and reduce the arterial wall distension, compliance, and overall arterial blood flow-regulating capacity. Consequently, arterial cushioning of aortic blood propulsion waves is diminished, increasing the distension and shear stress experienced by the arterioles and capillaries. With time, this pathologic, artery-driven mechanism contributes to remodelling of cerebral microvessels and the development of cerebral amyloid angiopathy (CAA)-related microvascular pathology characteristic in Alzheimer’s disease (AD)

We propose a disease model in which the loss of arterial vascular smooth muscle and degradation of arterial elastin are related to early Braak tau pathology (“Arterial Pathology Stage”) (Fig. 6). This arterial remodelling impairs the arterial wall distension dynamics that drive perivascular clearance and decreases the arteries’ aortic blood propulsion wave cushioning capacity, which increases the distension and shear stress on the downstream, relatively fragile arteriolar and capillary walls [51]. With time, this microvessel wall stress leads to pathologic arteriolar and capillary remodelling and, ultimately, contributes to CAA- and AD-related microvascular pathology (Fig. 6, “Microvascular Pathology Stage”). Thus, a combination of vessel-specific therapeutics that protect and improve cerebral vessel wall dynamics and, hence, cerebral perfusion/clearance, and therapeutics directed against neuronal or aggregated protein targets may hold more promise than AD monotherapy.

## Electronic supplementary material

Below is the link to the electronic supplementary material.

Supplementary material 1 (PPTX 2432 kb)

Supplementary material 2 (PPTX 8796 kb)

Supplementary material 3 (PPTX 646 kb)

Supplementary material 4 (PPTX 4762 kb)

Supplementary material 5 (PDF 357 kb)
