# Supplementary material for: Tau pathology-dependent remodelling of cerebral arteries precedes Alzheimer’s disease-related microvascular cerebral amyloid angiopathy
Source: Acta Neuropathol. 2016 Mar 17;131:737–52. doi: 10.1007/s00401-016-1560-2 (PMC4835519; doi:10.1007/s00401-016-1560-2)
Supplement: Supplementary file 1 — Supplementary material 1 (PPTX 2432 kb) [file 401_2016_1560_MOESM1_ESM.pptx]

## Slide 1
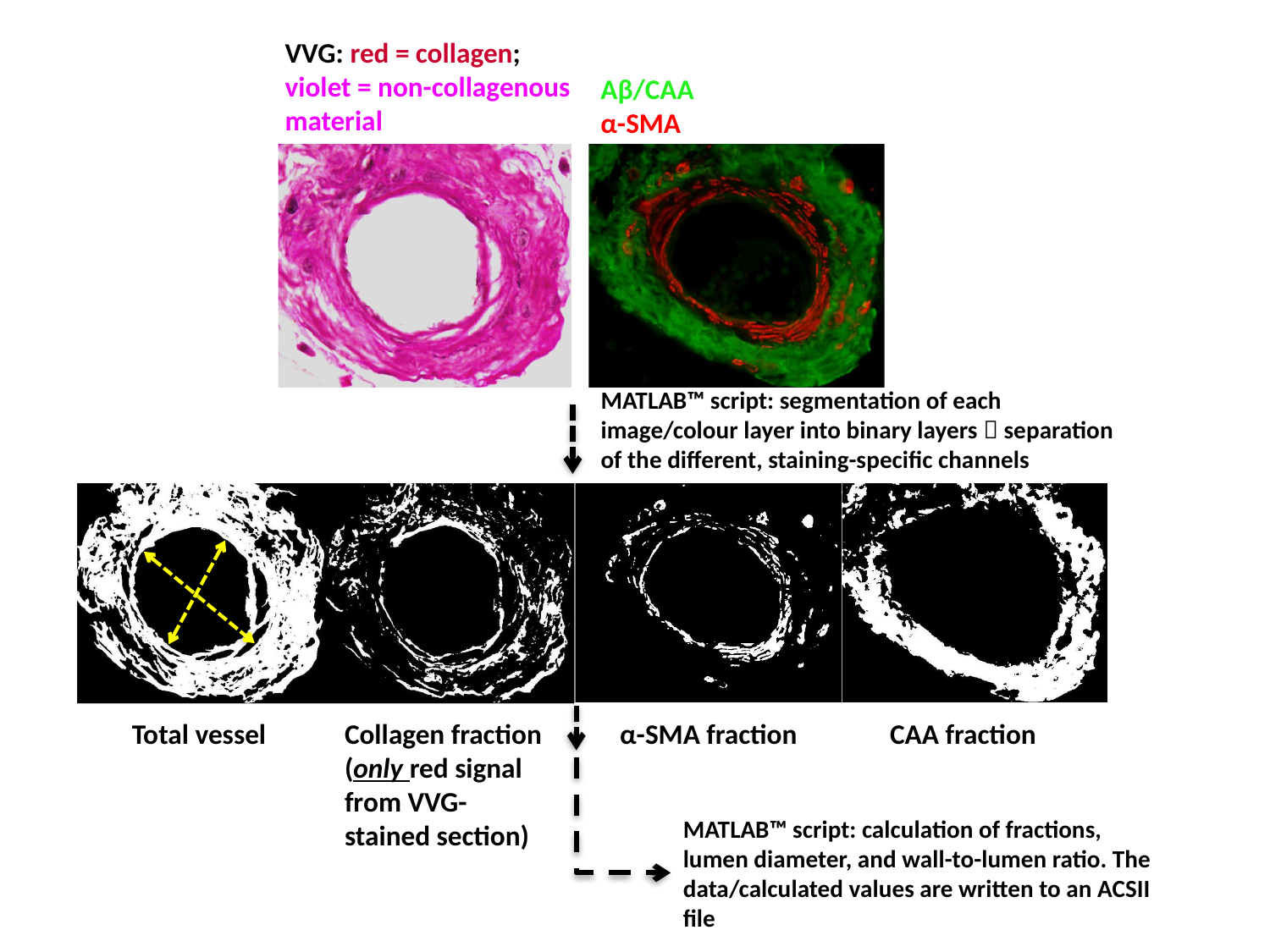

VVG: red = collagen;
violet = non-collagenous
material
Aβ/CAA
α-SMA
MATLAB™ script: segmentation of each image/colour layer into binary layers  separation of the different, staining-specific channels
Total vessel
Collagen fraction
(only red signal from VVG-stained section)
 α-SMA fraction
CAA fraction
MATLAB™ script: calculation of fractions, lumen diameter, and wall-to-lumen ratio. The data/calculated values are written to an ACSII file
