# Supplementary figures and images for: Tau pathology-dependent remodelling of cerebral arteries precedes Alzheimer’s disease-related microvascular cerebral amyloid angiopathy
Source: Acta Neuropathol. 2016 Mar 17;131:737–52. doi: 10.1007/s00401-016-1560-2 (PMC4835519; doi:10.1007/s00401-016-1560-2)

## Slide 1
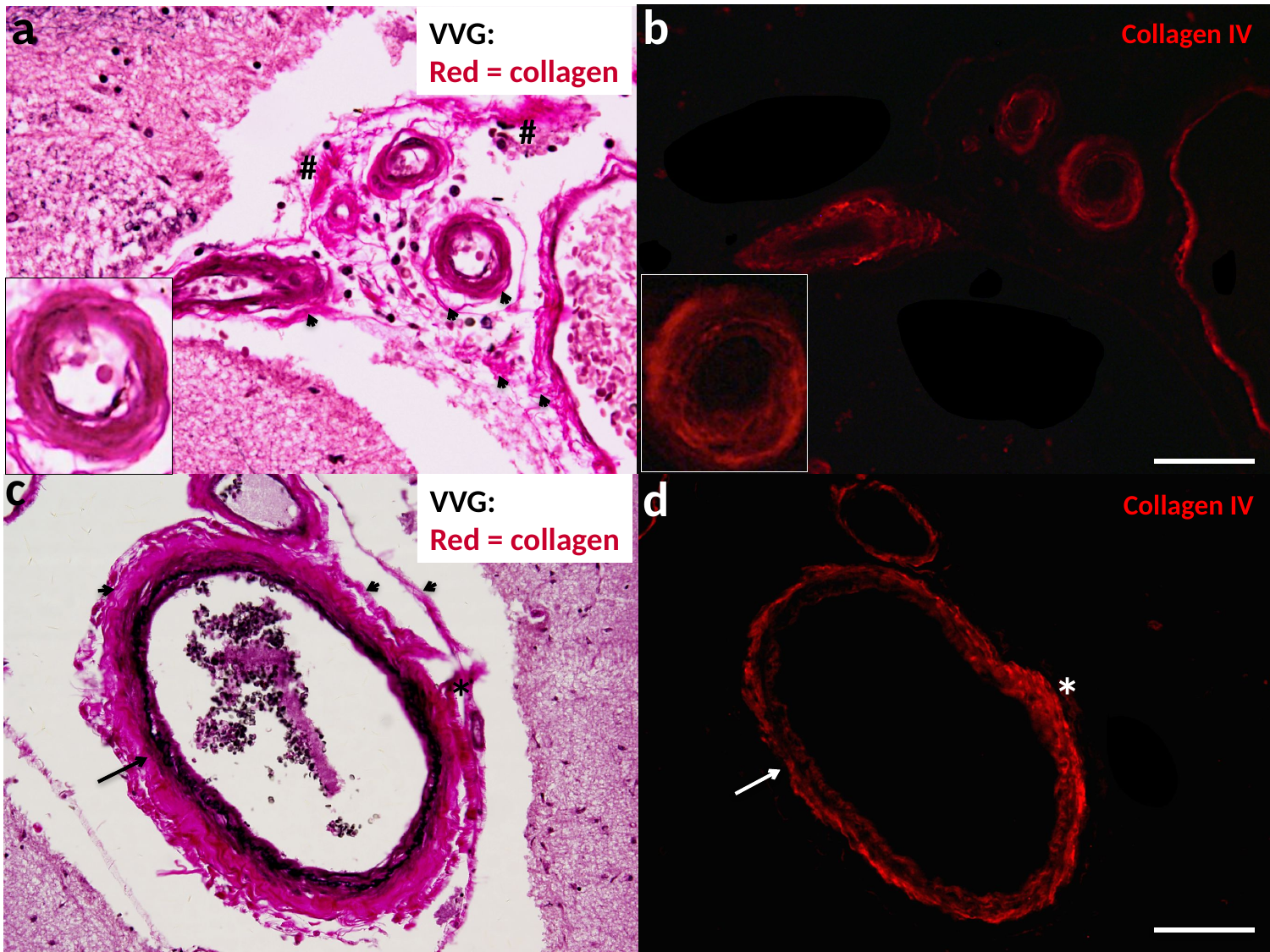

a
b
VVG:
Red = collagen
Collagen IV
#
#
c
d
VVG:
Red = collagen
Collagen IV
*
*

Supplement: Supplementary file 2 — Supplementary material 2 (PPTX 8796 kb) [file 401_2016_1560_MOESM2_ESM.pptx]

## Slide 1
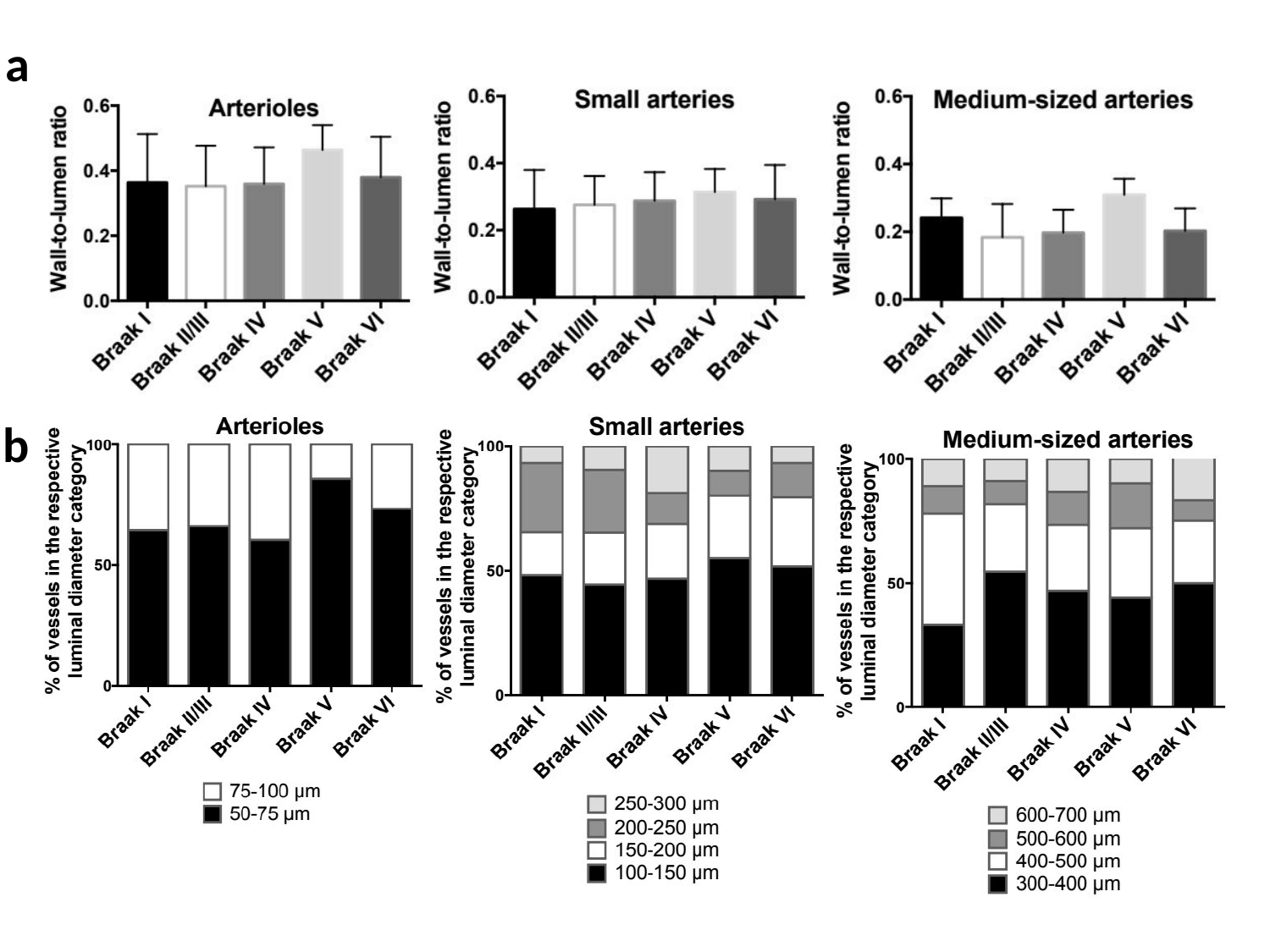

a
b

Supplement: Supplementary file 3 — Supplementary material 3 (PPTX 646 kb) [file 401_2016_1560_MOESM3_ESM.pptx]

## Slide 1
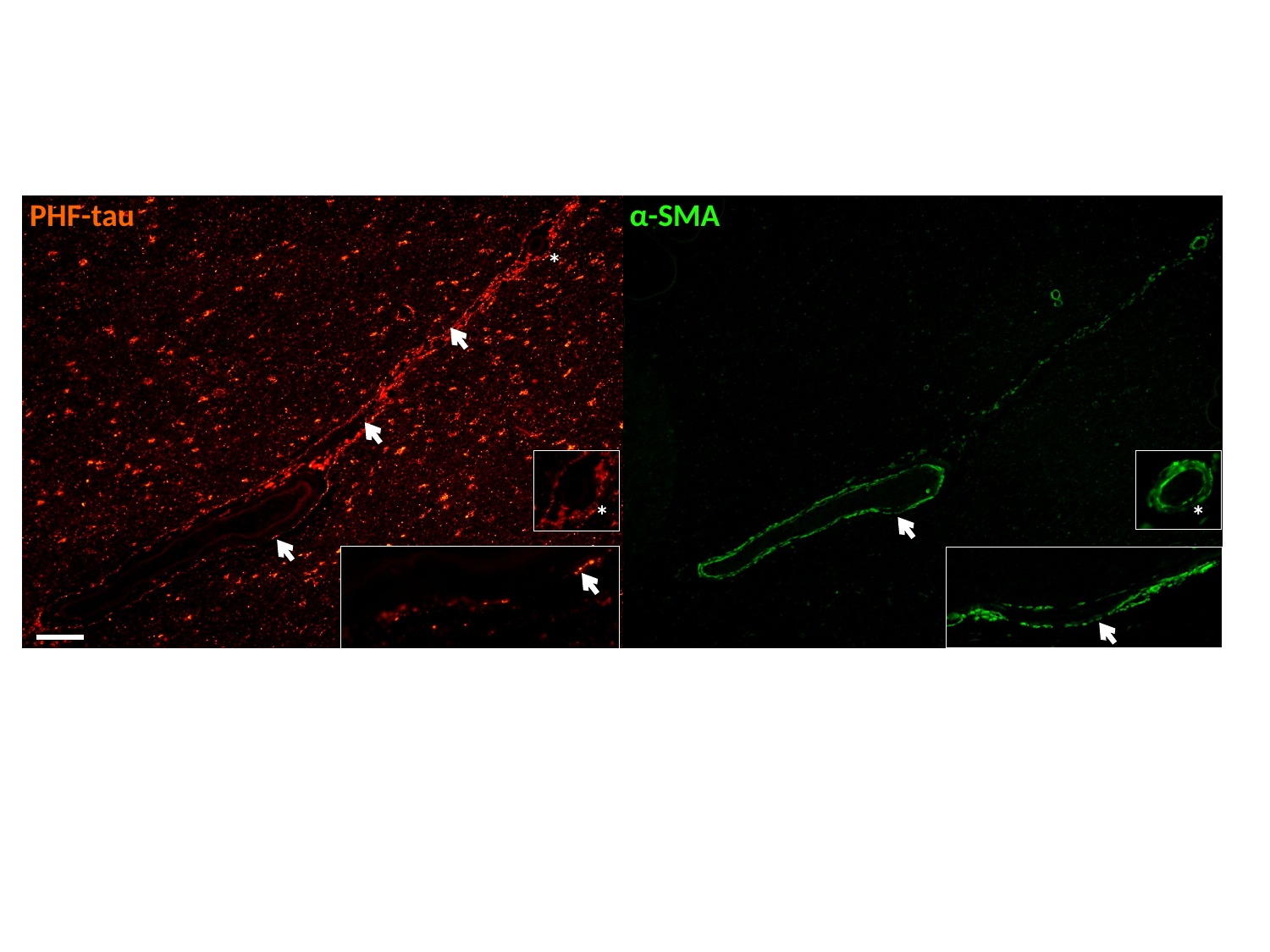

PHF-tau
α-SMA
*
*
*
*

Supplement: Supplementary file 4 — Supplementary material 4 (PPTX 4762 kb) [file 401_2016_1560_MOESM4_ESM.pptx]
