# Supplementary material for: Tau pathology-dependent remodelling of cerebral arteries precedes Alzheimer’s disease-related microvascular cerebral amyloid angiopathy
Source: Acta Neuropathol. 2016 Mar 17;131:737–52. doi: 10.1007/s00401-016-1560-2 (PMC4835519; doi:10.1007/s00401-016-1560-2)
Supplement: Supplementary file 5 — Supplementary material 5 (PDF 357 kb) [file 401_2016_1560_MOESM5_ESM.pdf]

Supplementary Material

Article title: Tau pathology-dependent remodelling of cerebral arteries precedes Alzheimer's disease-related microvascular cerebral amyloid angiopathy

Journal title: Acta Neuropathologica

Authors: Mario Merlini*, Debora Wanner and Roger M. Nitsch

***Corresponding author:** Center for Molecular Cardiology – Vascular Aging and Stroke, University of Zurich Schlieren Campus, Wagistrasse 12, CH-8952 Schlieren/Zurich, Switzerland

Tel.: +41 44 634 8878; E-mail address: mario.merlini@uzh.ch

Supplementary Materials and Methods

Histology

All reagents listed were purchased from Sigma-Aldrich (Basel, Switzerland) if not specified otherwise. The HIPP and GFM tissue blocks were cut in 5-μm thick sections and were mounted on SuperFrost® Plus microscope slides (VWR, Dietikon, Switzerland). Following standard deparaffinisation and rehydration steps, sections destined for immunohistochemical staining were processed as follows: antigen retrieval was achieved by first boiling the mounted brain sections in 0.1 M sodium citrate buffer containing 0.05% Tween®20 for 20 min and, following washing in phosphate-buffered saline (PBS, pH 7.4), by subsequently incubating the sections in 20 μg/mL proteinase K in Tris-EDTA buffer (50 mM Tris base, 1 mM EDTA, and 0.05% (v/v) Tween®20, pH 8.0) for 5 min at 37°C. The sections were washed with PBS, immuno-blocked in 10% donkey serum in Tris-buffered saline containing 0.2% Triton X-100 (TBS-T, pH 7.4) for 1 h at room temperature (RT), and were subsequently co-incubated with primary goat anti-alpha smooth muscle actin (α-SMA) antibody (ab21027; Abcam, Cambridge, UK) and primary mouse anti-Aβ (6E10, purified; Lucerna-Chem, Luzern, Switzerland) diluted 1:200 with 5% donkey serum in TBS-T for 2 h at RT. Following washing in PBS, the sections were co-incubated with donkey anti-mouse-Alexa488 and donkey anti-goat-Cy3 antibody (both from Jackson ImmunoResearch, Suffolk, UK) diluted 1:800 in TBST-T for 1 h at RT, washed with PBS, and coverslipped using Hydromount® mounting medium (National Diagnostics, Atlanta, GA, USA).

HIPP and GFM sections adjacent to the ones immunostained for α-SMA and Aβ were stained for collagen and elastin using the Verhoeff–van Gieson (VVG) stain.

The deparaffinised and rehydrated sections were incubated in Verhoeff stain (5% hematoxylin in absolute ethanol, 10% aqueous ferric chloride, 2% aqueous potassium iodide, and 1% aqueous iodine at a ratio of 2:1:1:1) for 1 h at RT. The sections were subsequently rinsed three times with tap water, differentiated in 2% aqueous ferric chloride for 2 min, washed with tap water, immersed in 5% aqueous sodium thiosulfate for 1 min, and washed with several changes of tap water for 5 min. The sections were subsequently counterstained in van Gieson's stain (1% aqueous acid fuchsin, saturated picric acid, and distilled water at a ratio of 0.3:1:1) for 6 min, quickly dehydrated through 95% ethanol and two changes of 100% ethanol, and cleared in two changes of xylene for 3 min each. The sections were coverslipped using Histomount® mounting medium (National Diagnostics, Atlanta, GA, USA).

Another series of adjacent HIPP and GFM sections was stained for neutrophil elastase. The deparaffinised and rehydrated sections were incubated in the antigen retrieval buffers, washed, and immuno-blocked as described above. Subsequently, the sections were incubated with primary rabbit anti-neutrophil elastase antibody (ab21595; Abcam, Cambridge, UK) diluted 1:100 with 5% donkey serum in TBS-T for 2 h at RT. Following washing in PBS, the sections were treated with 0.5% H_2O_2 in PBS for 30 min to block potential endogenous peroxidase activity, washed with PBS, incubated with secondary biotinylated donkey anti-rabbit antibody from the VectaStain® Elite staining kit (ReactoLab, Servion, Switzerland) for 45 min at RT, and further processed using the VectaStain® Elite staining kit to obtain Vector® SG substrate-stained (blue-grey) neutrophil elastase-positive cells per the manufacturer's instructions. The

sections were cleared and dehydrated to xylene and coverslipped using Histomount® mounting medium (National Diagnostics, Atlanta, GA, USA).

For determining the origin of neutrophil elastase in the vessel wall, i.e. whether it was neutrophil- and/or vascular smooth muscle-derived, co-localisation between neutrophil elastase and α-SMA was determined in confocal microscopy images of paraffin brain sections immuno-stained for neutrophil elastase and α-SMA as described above, followed by incubation with fluorescent secondary antibodies (neutrophil elastase: Cy3-conjugated donkey anti-rabbit antibody diluted 1:800, α-SMA: Alexa488-conjugated donkey anti-goat antibody diluted 1:800; Jackson ImmunoResearch, Suffolk, UK) for 1 h at RT. Nuclei were stained with DAPI.

Collagen stained by the van Gieson stain was compared to collagen stained by immunohistochemistry in a subset of brain sections. After antigen retrieval with citrate buffer and immuno-blocking as described above, brain sections were incubated with a mouse monoclonal antibody against human collagen IV (M 0785, diluted 1:25; DAKO, Gløstrup, Denmark) for 2 h at RT, followed by incubation with a donkey anti-mouse-Cy5 antibody (diluted 1:700; Jackson ImmunoResearch, Suffolk, UK) for 1 h at RT.

Accumulation of phosphorylated paired helical filament tau (PHF-tau) in the perivascular space of parenchymal vessels was detected with a mouse anti-PHF-tau antibody (AT8/MN1020, diluted 1:500; ThermoFisher Scientific, Reinach, Switzerland). Following antigen retrieval with citrate buffer and immuno-blocking as described above, HIPP brain sections were incubated with the anti-PHF antibody for 2 h at RT. Subsequently, the sections were stained

with a donkey anti-mouse-Cy3 antibody (diluted 1:700; Jackson ImmunoResearch, Suffolk, UK) for 1 h at RT.

Image acquisition

Microscopic images were acquired from arterioles and small arteries in the leptomeninges surrounding the GFM and HIPP, and from medium-sized leptomeningeal arteries in the sulci of the GFM and HIPP. Ten to fifteen vessels/brain region/subject from the VVG- and immunostained HIPP and GFM sections were imaged using a Leica DM4000B microscope (Leica, Wetzlar, Germany) equipped with an Olympus DP71 digital camera (Olympus, Volketswil, Switzerland) in bright field (neutrophil elastase-stained sections for vessel wall neutrophil elastase fraction measurements and VVG-stained sections) or fluorescent (Aβ- and α-SMA-stained sections) mode. For the latter, a dichroic filter cube with excitation and emission wavelength of 470/40 and 525/50, and 545/40 and 610/75 nm was used for Alexa488 and Cy3, respectively. Differentiation between the three vessel types was made based on vessel diameter, i.e. vessels with a diameter of 50–100, 100–300, and 300–700 μm were defined as arterioles, small arteries, and medium-sized arteries, respectively (Fig. 1a). Veins and venules were not imaged and were identified by their relatively small α-SMA-to-lumen ratio (Fig. 1a). Images were acquired using the image acquisition tool of the Visiopharm software (Visiopharm, Hørsholm, Denmark). All images were acquired in a blinded manner. Randomness of sample collection was achieved by overlaying the VVG-stained sections with a grid-engraved glass slide divided into numbered, 0.5 cm^2 squares. Starting at the top part of each section and following a counter clock-wise direction, the

leptomeningeal vessels present in each respective square were imaged at 100 × magnification (10 × objective) until 10–15 vessels/section were imaged. Using the same grid-engraved glass slide, the exact same vessels as those imaged on the VVG-stained sections were located on the adjacent fluorescently α-SMA- and Aβ-stained sections and were also imaged at 100 × magnification (10 × objective). Image acquisition parameters including fluorescence and bright field lamp intensity, shutter time, and camera exposure time were set once at the start of the image acquisition series and were kept constant for all sections/vessels and subjects.

Confocal z-stack images of the sections stained with DAPI and fluorescently labeled neutrophil elastase and α-SMA were acquired at an excitation wavelength of 405, 488, and 561 nm and an emission wavelength of 460, 520, and 570 nm for DAPI, Cy2, and Cy3, respectively, using a Leica SP8 confocal microscope (Leica, Wetzlar, Germany) in sequential imaging mode. The acquired images were converted into maximum z-stack projections in ImageJ (National Institutes of Health, Bethesda, MD, USA).

Image analysis: vessel wall ratio of Aβ, collagen, α-SMA, and neutrophil elastase and measurement of wall-to-lumen ratio and luminal diameter

The green fluorescent (Aβ) and red fluorescent (α-SMA) image channels were merged in the ImageJ software (National Institutes of Health, Bethesda, ML, USA). Stained cells and/or cellular material, e.g. red blood cells and collagen deposits, in the lumen of both the merged fluorescent and bright field VVG- and neutrophil elastase-stained vessels were removed using the ImageJ software to avoid false positive measurement hits in the subsequent quantification steps.

The vessel wall ratios of Aβ (CAA), collagen, α-SMA, and neutrophil elastase and the diameter of each vessel were quantified using an in-house established semi-automatic quantification script written in MATLAB™ (The MathWorks, Inc., Natick, MA, USA). The images of arterioles, small arteries, and medium-sized arteries acquired as described above were converted to an 8-bits (grey-scale) format, and the data contained in the 8-bits image layer were read in. Subsequently, the image layer was converted into a binary image and the intensity threshold was set to the value obtained during a previous manual calibration round in which the binary intensity of one randomly chosen image of a VVG-, Aβ-, α-SMA-, and neutrophil elastase-stained vessel/vessel type was set such that the collagen, elastin, CAA, α-SMA, and neutrophil elastase shown in the binary image matched with their morphology and density shown in the original, acquired image. This specific threshold value was implemented in the semi-automatic script and was kept constant for all images/vessels acquired from all subjects. The script segmented each image into its binary image, and further segmented each binary image into the respective binary fraction layer. The total vessel area, the wall fractions of interest, the vessel diameter, and wall-to-lumen ratio were calculated by the script from the data contained in the binary fraction layers. The respective vessel wall fractions were determined by dividing the calculated area of each vessel wall constituent by that of the total vessel area. The generated data were exported automatically to a data file sheet. The vessel wall area and the ratios counted by the script were displayed graphically in a final binary image to enable verification of the correctness of the data for each image (Supplementary Fig. 1).

Image analysis: elastin degradation

By comparing the elastin morphology of each imaged small artery and artery/brain region/subject to that of four reference arteries, elastin was classified by the experimenter as either not (score 0), mildly (score 1), moderately (score 2), or severely (score 3) degraded (Fig. 1b). The percentage of vessels in each degradation category was calculated according to $(n_{\text{vessel cat}})/(n_{\text{total vessel}}) \times 100$, where $n_{\text{vessel cat}}$ is the number of arteries or medium-sized arteries in the respective degradation category and Braak stage and $n_{\text{total vessel}}$ is the total number of arteries or medium-sized arteries analysed per Braak stage.

Supplementary Figure Legends

Supplementary Fig. 1 Schematic of the MATLAB™ image analysis method. Vessel images isolated from sections stained with the Verhoeff–van Gieson stain (VVG; red = collagen), adjacent sections double-stained immunohistochemically for amyloid β/cerebral amyloid angiopathy (CAA) and alpha-smooth muscle actin (α-SMA), and adjacent sections stained immunohistochemically for neutrophil elastase (not shown here) are segmented into binary images/layers, each representing the specific vessel wall component/fraction. Subsequently, the respective fraction values are calculated. The vessel diameter, lumen diameter (yellow arrows in the “Total vessel” image), and wall-to-lumen ratio are calculated as well

Supplementary Fig. 2 Comparison of collagen within the vessel wall stained with the van Gieson stain and with a collagen IV-specific antibody. Brain sections stained with the Verhoeff–van Gieson (VVG) stain show a collagen staining (**a** and **c**: bright red) within the vessel wall similar to that of adjacent brain sections stained with a monoclonal mouse anti-human collagen IV antibody (**b** and **d**). Compare the similarity in the low (arrows) and high (asterisks) signal intensity/staining between the bright red collagen stain in the VVG- (**c**) and collagen IV-stained image (**d**). Collagen unrelated to the vessel wall/collagen other than collagen IV present in the leptomeninges is also stained by the van Gieson stain (**a**, number signs). Non-collagenous material is indicated by arrowheads (VVG: **a** and **c**). The MATLAB™ script used to specifically segment the collagen fraction of images of vessels carefully cropped/separated from the total image that included the surrounding leptomeninges detects only the bright red-stained material (collagen). Scale bar in **b** = 50 µm, in **c** = 100 µm

Supplementary Fig. 3 Absence of Braak stage-dependent changes in the arteriolar and arterial wall-to-lumen ratio and luminal diameter. The wall-to-lumen ratio of leptomeningeal arterioles and small and medium-sized leptomeningeal arteries does not differ between the Braak stages analysed (**a**). The percentage of arterioles with the smallest luminal diameters measured is increased at Braak stage V and VI albeit non-significantly (**b**). A smaller shift in the luminal distribution range is observed for small arteries (>Braak stage III) and medium-sized arteries (>Braak stage I). The graphs represent the mean ± SE of 10–15 vessels/vessel category/subject of a total of 45 subjects

Supplementary Fig. 4 Accumulation of phosphorylated paired helical filament tau in the perivascular space of intraparenchymal vessels. Extensive accumulation of phosphorylated paired helical filament tau (PHF-tau) in the perivascular space of an intraparenchymal small and medium-sized artery in the hippocampus (border subiculum with CA1 region) of a Braak stage II subject without cerebral amyloid angiopathy (PHF-tau staining: arrowheads and asterisk). Intraparenchymal perivascular accumulation of PHF-tau is paralleled by alpha smooth muscle actin (α-SMA) loss (α-SMA staining: arrowheads and asterisk). Images are from consecutive brain sections. Scale bar = 100 μm
